# Gallium-68 prostate-specific membrane antigen ([^68^Ga]Ga-PSMA-11) PET for imaging of thyroid cancer: a feasibility study

**DOI:** 10.1186/s13550-020-00720-3

**Published:** 2020-10-22

**Authors:** Courtney Lawhn-Heath, Sue S. Yom, Chienying Liu, Javier E. Villanueva-Meyer, Maya Aslam, Raven Smith, Manpreet Narwal, Roxanna Juarez, Spencer C. Behr, Miguel Hernandez Pampaloni, Jason W. Chan, Christine M. Glastonbury, Thomas A. Hope, Robert R. Flavell

**Affiliations:** 1 Department of Radiology and Biomedical Imaging, University of California San Francisco, 185 Berry Street, Suite 350, Lobby 6, Box 0946, San Francisco, CA 94143 USA; 2 Department of Radiation Oncology, University of California San Francisco, San Francisco, CA USA; 3 Department of Medicine, Division of Endocrinology, University of California San Francisco, San Francisco, CA USA; 4 Department of Pharmaceutical Chemistry, University of California San Francisco, San Francisco, CA USA

**Keywords:** Thyroid cancer, PSMA PET, PSMA therapy, Theranostics, PET/MRI

## Abstract

**Background:**

Prostate-specific membrane antigen (PSMA) is expressed in the microvasculature of thyroid cancer. This suggests the potential use of PSMA as a diagnostic agent in patients with aggressive forms of thyroid cancer. The purpose of the current study was to determine the feasibility and utility of [^68^Ga]Ga-PSMA-11 PET/MRI in thyroid cancer patients.

**Methods:**

Eligible patients for this prospective pilot study were adults with a history of pathology-proven thyroid cancer who had abnormal radiotracer uptake on an 2-[^18^F]FDG PET and/or ^131^I scintigraphy performed in the 12 months prior to study enrollment. Patients underwent a [^68^Ga]Ga-PSMA-11 PET/MRI, and comparison was made to the prior qualifying 2-[^18^F]FDG PET CT/MRI for lesion location and relative intensity.

**Results:**

Twelve patients underwent [^68^Ga]Ga-PSMA-11 PET/MRI, one of which was excluded from analysis due to debulking surgery prior to the PSMA PET. Of the remaining patients, 7/11 had differentiated disease (3 papillary, 2 follicular, 2 Hurthle cell) and 4/11 had dedifferentiated disease (2 poorly differentiated papillary, 2 anaplastic). Out of 43 lesions, 41 were visually 2-[^18^F]FDG positive (uptake greater than background, detection rate 95.3%) and 28 were PSMA positive (uptake greater than background, detection rate 65.1%). Uptake was heterogeneous between patients, and in some cases within patients. 3/11 patients (1 poorly differentiated papillary, 2 follicular) had PSMA uptake which was greater than FDG uptake. For the remaining 8 patients, 2-[^18^F]FDG uptake was greater than PSMA. Using one eligibility guideline in the prostate cancer literature for PSMA radioligand therapy (RLT), 8/11 could be considered eligible for possible future PSMA RLT. This was not predictable based on thyroid cancer subtype.

**Conclusions:**

[^68^Ga]Ga-PSMA-11 PET demonstrated lower detection rate when compared to 2-[^18^F]FDG PET for thyroid cancer lesion visualization. Thyroid cancer subtype alone may not be sufficient to predict PSMA uptake, and radiotracer uptake may vary between patients and even within patients.

## Background

Thyroid cancer is the most common endocrine malignancy, with approximately 62,000 new cases per year in the USA [1].The vast majority of thyroid cancer patients respond favorably to surgery and risk-adapted postoperative therapy with thyroid hormone suppression and radioactive iodine (RAI) therapy, with external beam radiation therapy added in select high-risk situations [2, 3]. The best response is seen in differentiated thyroid cancers (DTC): papillary thyroid cancer (PTC), follicular thyroid cancer (FTC), and Hurthle cell carcinoma (HCC). However, a subset of patients with aggressive and less differentiated thyroid cancer refractory to RAI therapy ultimately succumb to their illness, with approximately 2000 deaths per year in the US [4]. While DTC can become resistant to RAI therapy, resistance is most commonly seen in dedifferentiated subtypes (such as poorly differentiated papillary thyroid cancer (PDPTC) and anaplastic thyroid cancer (ATC). For patients with disease that is not amendable to surgery and/or becomes iodine refractory (does not take up iodine in one or more lesions, or progresses despite RAI therapy [5]), treatment options are limited. The prognosis for these patients remains poor. Therefore, there is an unmet clinical need for improved therapies for these patients with aggressive forms of thyroid cancer.

Agents targeting prostate-specific membrane antigen (PSMA) are under investigation for both imaging and therapy of prostate cancer [6, 7].[^68^Ga]Ga-PSMA-11 is currently under consideration for FDA approval in biochemically recurrent prostate cancer, and the phase 3 VISION trial evaluating safety and efficacy of [^177^Lu]Lu-PSMA-617 therapy is currently underway [8]. However, despite its name, PSMA expression is not specific to prostate, and is seen in a variety of normal tissues and cancers [9–11]. In particular, multiple recent reports have demonstrated that PSMA directed agents demonstrate uptake in thyroid cancer [12–15]. Specifically, histological studies have verified expression of PSMA in the microvasculature of thyroid cancers [16–18]. This evidence suggests the potential use of PSMA PET as a diagnostic agent in patients with thyroid cancer. A recent pilot study [15] demonstrated the potential utility of PSMA PET in selecting papillary thyroid cancer patients for possible PSMA radioligand therapy, but this has not been evaluated in other thyroid cancer subtypes. The purpose of the current study was to determine the feasibility and utility of [^68^Ga]Ga-PSMA-11 PET/MRI in thyroid cancer patients, including both differentiated and dedifferentiated subtypes.

## Methods

### Patient selection

This was a single-center prospective, open-label pilot study approved by the UCSF institutional review board (IRB number 17-23649). All patients provided written informed consent prior to their participation in the study.

Eligible patients were adults with a history of pathology-proven thyroid cancer who had abnormal radiotracer uptake on an 2-[^18^F]FDG PET and/or [^123^I]iodide/[^131^I]iodide scintigraphy performed in the 12 months prior to study enrollment. Thyroid cancer subtype was determined by the most recent biopsy or surgical specimen prior to study enrollment. Patients were excluded from the study if serum creatinine was > 3.0 mg/dL or if they were pregnant, breastfeeding, unable to have placement of an intravenous line, or unable to undergo PET/MRI due to weight, claustrophobia, or inability to lie still for the duration of the exam. Patients were monitored for adverse events while in the department by nursing staff.

### Imaging protocol: PSMA PET

[^68^Ga]Ga-PSMA-11 was produced under good manufacturing practices (GMP) as previously described [19] using an ITG germanium-gallium generator and an iQs fluidic labeling module (ITG, Gärching/Munich, Germany). A median of 73 min (range 53–121 min) following intravenous injection of median 207.2 MBq (range 173.9–299.7 MBq, median 5.6 mCi, range 4.7–8.1 mCi) [^68^Ga]Ga-PSMA-11, patients underwent PET/MRI (3.0 T time-of-flight Signa PET/MRI, GE Healthcare, Waukesha, WI). Targeted MR imaging of the neck was performed in two stations (upper and lower neck) using axial pre-gadolinium T1 weighted, T2 weighted, diffusion weighted, and post-gadolinium T1 weighted sequences. Whole-body PET and whole-body MRI were simultaneously acquired for 3 min at each bed position with concurrent axial T1 and T2 weighted sequences, from the vertex to mid-thighs with arms overhead if tolerated. PET data was reconstructed using time-of-flight, OSEM with two iterations and 28 subsets. A matrix size of 256 × 256 was used, with 600 × 250 mm field of view, and 2.8 mm slice thickness. As has been described [20], attenuation correction with a standard two-point Dixon acquisition converted into an attenuation map was performed.

### Prior imaging techniques: ^18^FDG PET

For scans performed at our institution, patients fasted for at least 6 h. 55–70 min after the IV administration of 262.7–377.4 MBq (7.1–10.2 mCi) 2-[^18^F]FDG, non-contrast CT was performed from vertex to thighs. This was followed by emission PET imaging from vertex to thighs for 4 min per bed position. PET images were corrected for attenuation using the CT transmission data. Some prior ^18^FDG PET studies were from outside institutions with unknown acquisition techniques.

### Prior imaging techniques: [^123^I]Iodide/[^131^I]Iodide scan

Patients imaged received [^131^I]Iodide scans post-therapy, with a planning [^123^I]Iodide scan prior to treatment. For ^123^I scans performed at our institution, anterior and posterior planar images of the whole body were acquired 24 h after oral admin of 74–129.5 MBq (2–3.5 mCi) [^123^I]Iodide. For [^131^I]Iodide scans performed at our institution, imaging was acquired 7 days after treatment with 2.6–5.41 GBq (72.0–146.1 mCi) [^131^I]Iodide and consisted of anterior and posterior whole-body planar images supplemented by SPECT/CT images of the regions of the body where abnormal uptake was identified by the supervising nuclear radiologist. Some prior [^123^I]Iodide/[^131^I]Iodide studies were from outside institutions with unknown acquisition techniques.

### Image analysis

[^68^Ga]Ga-PSMA-11 PET/MRI images were evaluated by two dual board-eligible (CLH and RJ) and one dual board-certified (RF) nuclear medicine physicians and radiologists. Comparison was made to the prior qualifying 2-[^18^F]FDG PET CT/MRI and/or [^123^I]Iodide/[^131^I]Iodide scintigraphy for lesion locations and relative intensity. CLH and RJ measured the SUVmax of all avid lesions on both the [^68^Ga]Ga-PSMA-11 PET/MRI and the prior 2-[^18^F]FDG PET CT/MRI, as well as background SUVmax in the mediastinum using a volume of interest (VOI) measuring 2 cm in diameter. If a lesion was only avid above background on one PET scan (either the PSMA or FDG PET but not both), a VOI was placed in the expected region of the lesion based on the CT/MRI. If there was no CT/MR correlate to guide VOI placement, the expected location of the lesion was approximated by comparing to the location of the avid focus on the other PET study.

Dedicated neck MRI images were evaluated by a board-certified neuroradiologist (JVM) for the presence of locally recurrent disease, metastatic disease, and post-treatment changes.

## Results

### Patient characteristics

Twelve patients underwent [^68^Ga]Ga-PSMA-11 PET/MRI, with baseline characteristics and history as per Table 1. One patient with extensive cervical disease on qualifying FDG PET/CT underwent surgical debulking prior to undergoing [^68^Ga]Ga-PSMA-11 PET/MRI and was therefore excluded from the analysis due to lack of comparability. Of the remaining 11 patients, 7 had DTC: 3 PTC, 2 FTC, and 2 HCC. Four had dedifferentiated thyroid cancer: 2 PDPTC and 2 ATC. There were 43 lesions present across all 11 patients (33 lesions in DTC and 10 in dedifferentiated subtypes). Patients were a median age of 65.5 years (range 47–80 years), and a median of 3 years from initial diagnosis (range 0–28 years) at the time of [^68^Ga]Ga-PSMA-11 PET/MRI.[^68^Ga]Ga-PSMA-11 PET/MRI was performed a median of 1.8 months (range 0.4–11.4 months) from most recent 2-[^18^F]FDG PET (in 10/11 patients) or [^123^I]Iodide/[^131^I]Iodide scan (in 1/11 patients), with 9/11 patients undergoing [^68^Ga]Ga-PSMA-11 PET/MRI within 3 months of most recent imaging (Table 1). None of the 11 included patients received any new treatments or treatment changes between qualifying imaging and [^68^Ga]Ga-PSMA-11 PET/MRI. All patients had metastatic disease (10/11 distant, 1/11 local cervical). 2/11 patients’ 2-[^18^F]FDG exams were from outside institutions. Of the 8/11 patients for whom prior [^123^I]Iodide/[^131^I]Iodide studies were available, 2/8 patients’ [^123^I]Iodide/[^131^I]Iodide studies were from outside institutions. The remaining 6 patients’ most recent radioactive iodine studies were all [^131^I]Iodide post-therapy scans ranging in dates from 2012 to 2019.

**Table 1:** Patient characteristics at the time of [^68^Ga]Ga-PSMA-11 scan

Patient #	Age	Sex	Thyro-globulin (ng/mL); Ab +/−	TSH (mIU/ L)	Tumor type	Years since diagnosis	Treatment history	Years Since RAI; EBRT	I-123/131 uptake on most recent scan?	Months to PSMA PET
1	78	M	17; −	0.36	PTC	14	S, RAIx1, EBRT	6; 1	No	3.0
2	58	F	520.2; +	0.01	HCC	5	S, RAIx2, EBRT	3; 1	No	1.8
3	80	M	22.0; −	0.11	PDPTC	28	S	n/a; n/a	No	2.4
4	79	M	245.1; +	2.7	ATC	< 1	S	n/a; n/a	Unknown	0.4
5	72	F	1771.0; −	n/a	FTC	5	S, RAIx2	4; n/a	No	1.8
6	65	F	2125.8;−	0.01	PDPTC	3	S, EBRT	n/a; 1	No	3.0
7	66	F	7.3; −	0.11	PTC	3	S, RAIx1, EBRT	3; 3	No	5.3
8	46	F	0.1; −	12.1	ATC	< 1	S, RAIx1	0.5; n/a	No	1.2
9	62	M	0.1; +	0.37	PTC	2	S, RAIx2	1; n/a	No	0.7
10	47	F	20,540.0; −	0.01	FTC	< 1	S	n/a; n/a	Yes*	11.4
11	54	M	15.6; −	0.13	HCC	8	S, RAIx3, EBRT	4; 2	Unknown	1.5

Time to PSMA PET: months from most recent 2-[^18^F]FDG PET or [^123^I]Iodide/[^131^I]Iodide scan to [^68^Ga]Ga-PSMA-11 PET

*Ab* anti-thyroglobulin antibodies, *TSH* thyroid stimulating hormone, *PTC* papillary thyroid carcinoma, *HCC* Hurthle cell carcinoma, *PDPTC* poorly differentiated papillary thyroid carcinoma, *ATC* anaplastic thyroid carcinoma, *FTC* follicular thyroid carcinoma, *S* surgery, *RAI* radioactive iodine therapy, *EBRT* external beam radiation therapy, *Chemo* chemotherapy

^*^Patient 10 was planned for RAI which was performed approximately 1 month after imaging workup (including PSMA PET)

### Imaging findings

 Side-by-side maximum intensity projection (MIP) images of each patient’s PSMA and FDG PET exams, grouped by cancer subtype, can be found in Additional file 1: Figures S1–S5. 8 out of 11 patients had positive disease (uptake visually greater than background) on PSMA PET, while 9 out of 11 patients had positive disease on FDG PET. Disease locations and median lesion SUVmax are listed in Table 2. Out of 43 lesions, 41 were visually FDG positive (detection rate 95.3%) and 28 were PSMA positive (detection rate 65.1%). The 2 FDG negative lesions (both osseous) were PSMA positive in an FTC patient. Overall median lesion SUVmax was 9.0 for FDG PET (range 2.6–28.4) and 8.5 for PSMA PET (range 1.6–27.8). Median SUVmax was substantially lower for lung lesions [median SUVmax 4.2 (range 2.5–19.1) for FDG PET and 2.6 (range 1.2–4.5) for PSMA PET] compared to other locations, and highest for extrapulmonary soft tissue metastases (median SUVmax 14.2, range 3.5–31.4) for FDG PET and 15.8 (range 6.2–27.7) for PSMA PET (Table 3).

**Table 2:** Metastatic disease locations and median lesion SUVmax for each patient

Patient #	Tumor type	Disease locations	PSMA median SUVmax (range)	FDG median SUVmax (range)	Neck MRI findings	Liver SUVmax	PSMA > liver + No FDG/PSMA discordance?
1	PTC	B	9.0 (1.0–10.1)	21.1 (4.1–28.4)	Post-tx, bone mets (P,F)	7.2	Yes
2	HCC	B, ST	11.0 (3.4–27.8)	11.6 (4.7–31.3)	Post-tx, bone mets (F)	13.2	Yes
3	PDPTC	ST	4.5 (1.9–6.2)	5.3 (4.1–10.3)	Post-tx (non-avid)	5.6	Yes
4	ATC	ST, TB	3.3 (1.6–6.0)	14.3 (4.2–19.1)	Post-tx, “treated” residual TB tissue (P,F)	9.5	No
5	FTC	B	15.0 (15.0–15.0)	3.5 (3.5–3.5)	Post-tx (non-avid)	6.5	Yes
6	PDPTC	LN, ST, TB	8.5 (4.3–12.6)	4.1 (2.6–5.6)	Post-tx (P,F)	10.2	Yes
7	PTC	ST	2.7 (2.7–2.7)	17.0 (17.0–17.0)	Post-tx (non-avid)	9.2	No
8	ATC	TB	6.0 (5.7–6.3)	14.1 (13.8–14.3)	Post-tx, TB soft tissue (P,F)	5.7	Yes
9	PTC	LN	2.5 (2.0–11.4)	5.9 (5.3–6.9)	Post-tx, decreasing cervical LN (F)	7.1	Yes
10	FTC	B	13.8 (9.2–18.3)	8.0 (7.4–8.7)	Post-tx (non-avid)	5.4	Yes
11	HCC	ST	2.8 (2.5–3.1)	4.3 (3.9–4.7)	Post-tx (non-avid)	13.9	No

*B* bones, *ST* soft tissues, *TB* thyroid bed, *LN* lymph nodes, *Post-tx* post-treatment changes (thyroidectomy, scarring, and/or lymph node dissection). The last column refers to PSMA RLT eligibility criteria used in a recent phase 2 trial of PSMA RLT in prostate cancer [21]. Reasons for lack of eligibility: patient 4—FDG avid lung metastases not seen on PSMA; patient 8—FDG avid lung metastases with minimal uptake on PSMA; patient 12—FDG avid lung metastases not seen on PSMA

**Table 3:** Median lesion SUVmax and discordant lesions by location

Location	Median PSMA SUVmax (range)	Median FDG SUVmax (range)	Number of discordant lesions/total lesions
Bones	10.1 (3.5–18.3)	11.7 (3.9–28.4)	8/19 (PSMA −, FDG +), 2/19 (FDG−, PSMA +)
Lymph nodes	7.8 (2.0–12.6)	5.9 (5.3–10.4)	4/5 (PSMA −, FDG +)
Lungs	2.6 (1.6–4.5)	4.2 (2.5–19.1)	2/5 (PSMA −, FDG +)
Soft tissue: other	15.8 (6.2–27.7)	14.2 (3.5–31.4)	0/6
Thyroid bed	6.0 (5.7–6.3)	14.3 (13.8–14.3)	1/3 (PSMA −, FDG +)

Soft tissues are subdivided into lungs (most common) and “other.” “Other” locations consisted of: scalp, dura, adrenal, paraspinous tissues, and pancreas

**Fig. 1:**
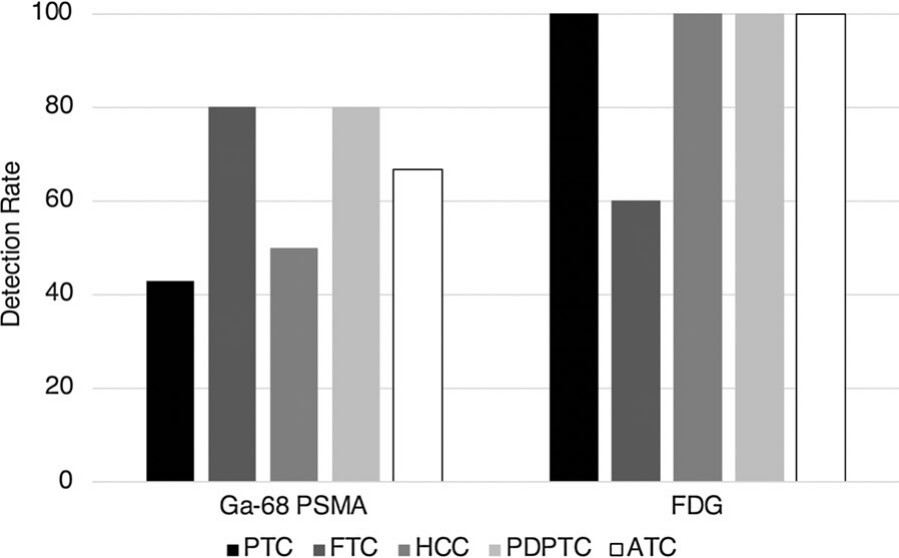
Detection rate of [^68^Ga]Ga-PSMA-11 and 2-[^18^F]FDG PET by thyroid cancer subtype. PTC: papillary thyroid carcinoma. FTC: follicular thyroid carcinoma. HCC: Hurthle cell carcinoma. PDPTC: poorly differentiated papillary thyroid carcinoma. ATC: anaplastic thyroid carcinoma

Detection rates for all DTC in the cohort were 93.8% for FDG PET and 53.1% for PSMA PET. For DTC, median lesion SUVmax was 9.0 for FDG PET (range 3.5–28.4) and 9.2 for PSMA PET (range 2.0–27.8). Detection rates for all dedifferentiated thyroid cancers in the cohort were 100% for FDG PET and 72.7% for PSMA PET. For dedifferentiated thyroid cancers, median lesion SUVmax was 8.0 for FDG PET (range 2.6–19.1) and 5.1 for PSMA PET (range 1.6–12.6). Detection rates for individual cancer subtypes are listed in Fig. 1. For PSMA, detection rate was highest for FTC (80.0%) and PDPTC (80.0%), although the small number of patients in each group limits this analysis.

**Fig. 2:**
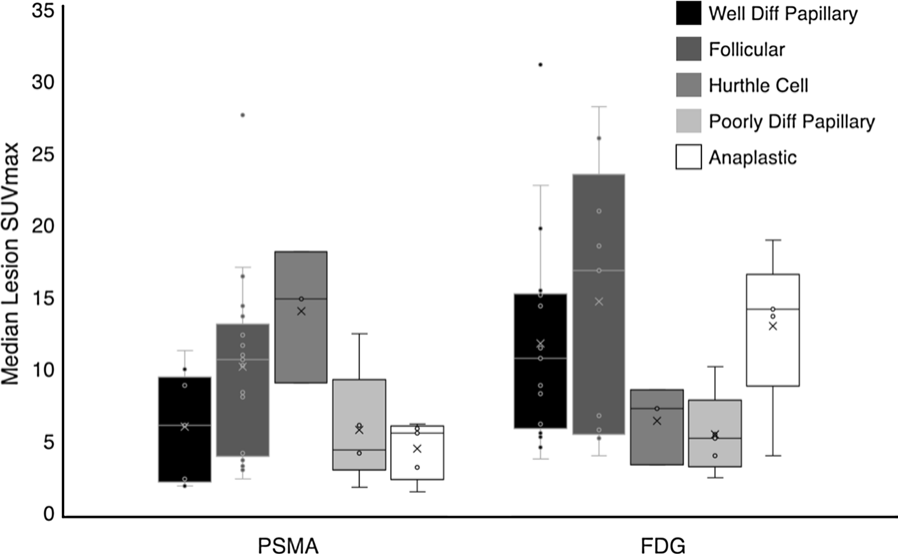
Box and whisker plot of Median SUVmax for all lesions by thyroid cancer subtype. Diff = differentiated

**Fig. 3:**
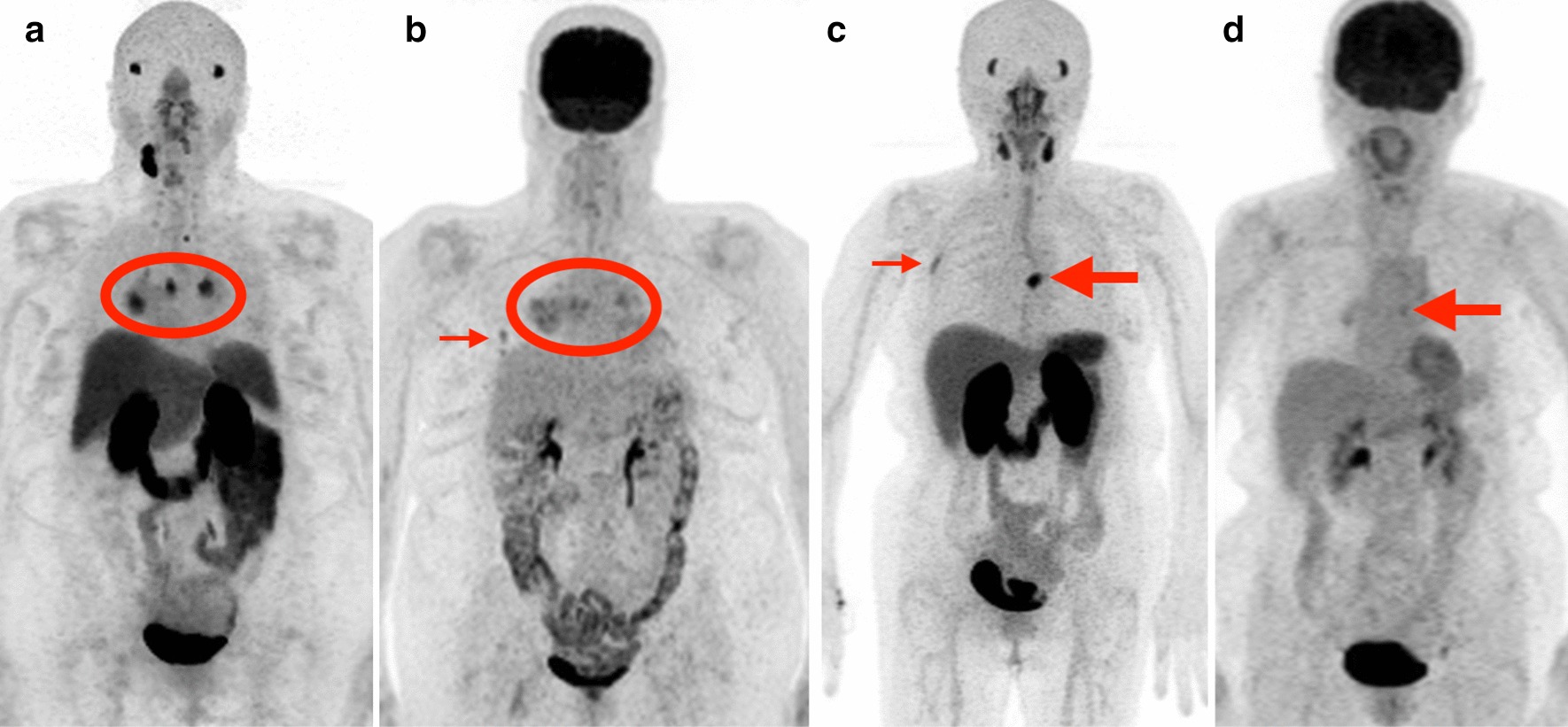
Patients with metastatic thyroid cancer with greater PSMA uptake than FDG uptake: examples. **a**, **b** [^68^Ga]Ga-PSMA-11 PET MIP (**a**) and 2-[^18^F]FDG PET MIP (**b**) in a patient (Patient 6) with poorly differentiated papillary thyroid carcinoma. Bilateral hilar and mediastinal metastases (circled) are more avid on PSMA (median SUVmax 8.5) than FDG (median SUVmax 4.1). However, a small right middle lobe metastasis seen on FDG (B, small arrow) is not visible on PSMA. C–D: [^68^Ga]Ga-PSMA-11 PET MIP (**c**) and 2-[^18^F]FDG PET MIP (**d**) in a patient (Patient 5) with follicular thyroid carcinoma. A left 8th costovertebral junction metastasis (large arrows) is more avid on PSMA (SUVmax 15.0) than on FDG (SUVmax 3.5). In addition, a focus of uptake in the lateral right 4th rib (**c**, small arrow) is only visible on PSMA PET and may represent an additional site of metastatic disease

Overall, tumor radiotracer uptake was heterogeneous within cancer subtypes and even within individual patients. Figure 2 displays median lesion SUVmax on PSMA versus FDG PET by cancer subtype. In some patients, PSMA uptake was higher than FDG uptake. For example, Patient 6 with PDPTC had a higher median SUVmax in mediastinal and bilateral hilar metastases on PSMA PET (8.5) than on FDG (4.1) (Fig. 3a, b). However, there was a small right middle lobe metastasis which was only seen on FDG PET and not PSMA PET. Interestingly, the other PDPTC patient (Patient 3) did not demonstrate a higher median lesion SUVmax on PSMA PET (4.5) compared to FDG PET (5.3). Like Patient 6 with PDPTC, Patient 5 with FTC also demonstrated higher median lesion SUVmax on PSMA PET (15.0) compared to FDG (3.5) (Fig. 3c, d). In addition, a right fourth rib metastasis was only seen on PSMA PET. The other FTC patient (Patient 10) also demonstrated higher PSMA than FDG uptake in two metastases, but substantially lower PSMA uptake than FDG uptake in a T10 metastasis (PSMA SUVmax 7.4; FDG SUVmax 18.3, Additional file 1: Figure 2C, D).

**Fig. 4:**
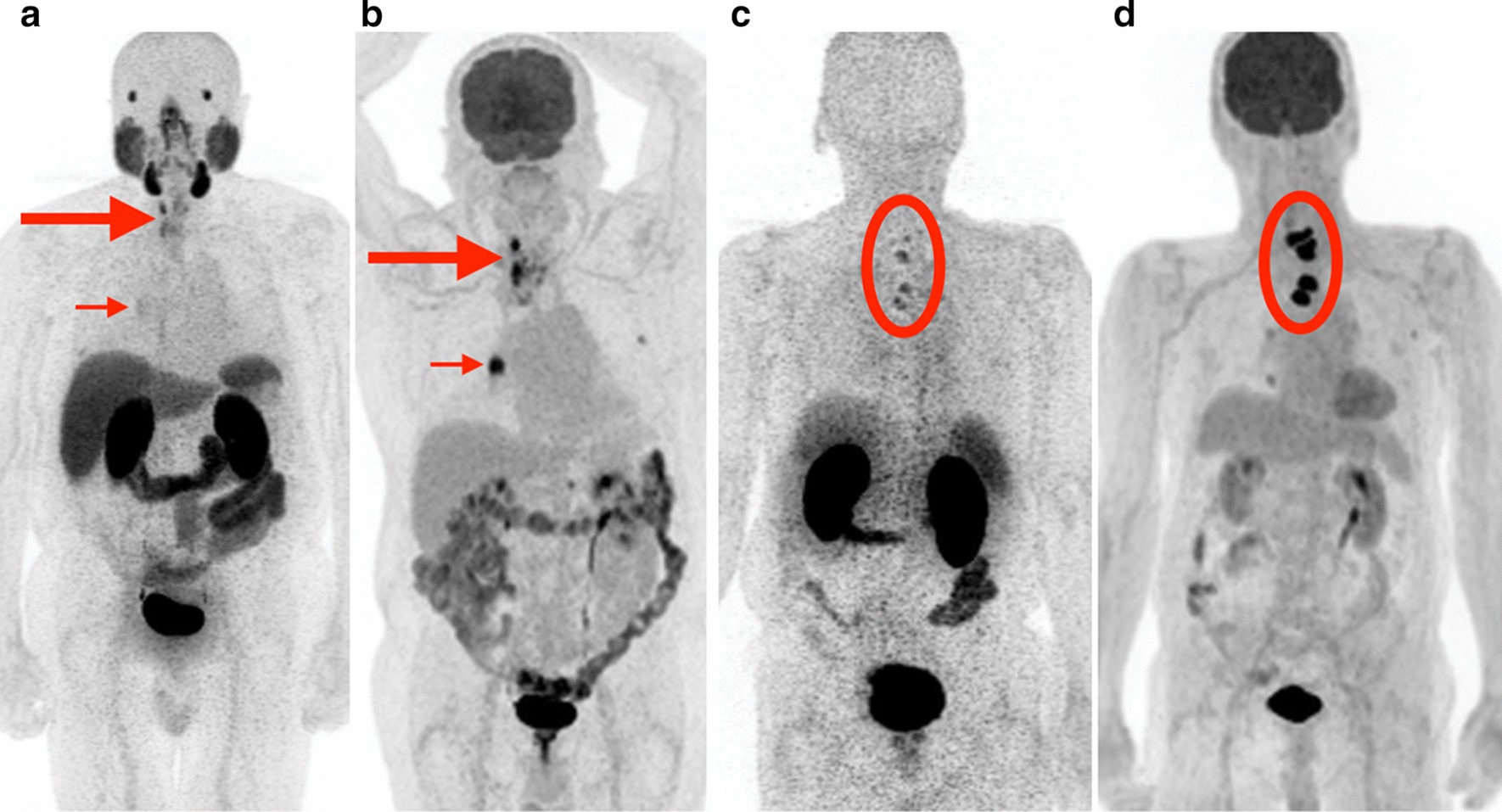
Patients with metastatic thyroid cancer with greater FDG uptake than PSMA uptake: examples. **a**, **b** [^68^Ga]Ga-PSMA-11 PET MIP (**a**) and 2-[^18^F]FDG PET MIP (**b**) in a patient (Patient 4) with anaplastic thyroid carcinoma. Thyroid bed recurrence (large arrows) and right hilar nodal metastasis (small arrows) are more avid on FDG (median SUVmax 14.3) than PSMA (median SUVmax 3.3). **c**, **d** [^68^Ga]Ga-PSMA-11 PET MIP (**c**) and 2-[^18^F]FDG PET MIP (**d**) in a patient (Patient 1) with papillary thyroid carcinoma. Multiple lower cervical and upper thoracic vertebral body metastases (circled) are more avid on FDG (median SUVmax 21.1) than on PSMA (median SUVmax 9.0)

For most patients (8/11), lesion FDG uptake was higher than PSMA uptake. For example, Patient 4 with ATC had much higher tumor FDG uptake (median SUVmax 14.3) than PSMA uptake (SUVmax 3.3, Fig. 4a, b), as did Patient 1 with PTC (median FDG SUVmax 21.1; median PSMA SUVmax 9.0, Fig. 4 c, d). Although 8/11 patients had higher FDG uptake than PSMA uptake, lesions were still considered positive (that is, visually greater than or equal to blood pool) on PSMA PET in 5 of the 8 patients.

**Fig. 5:**
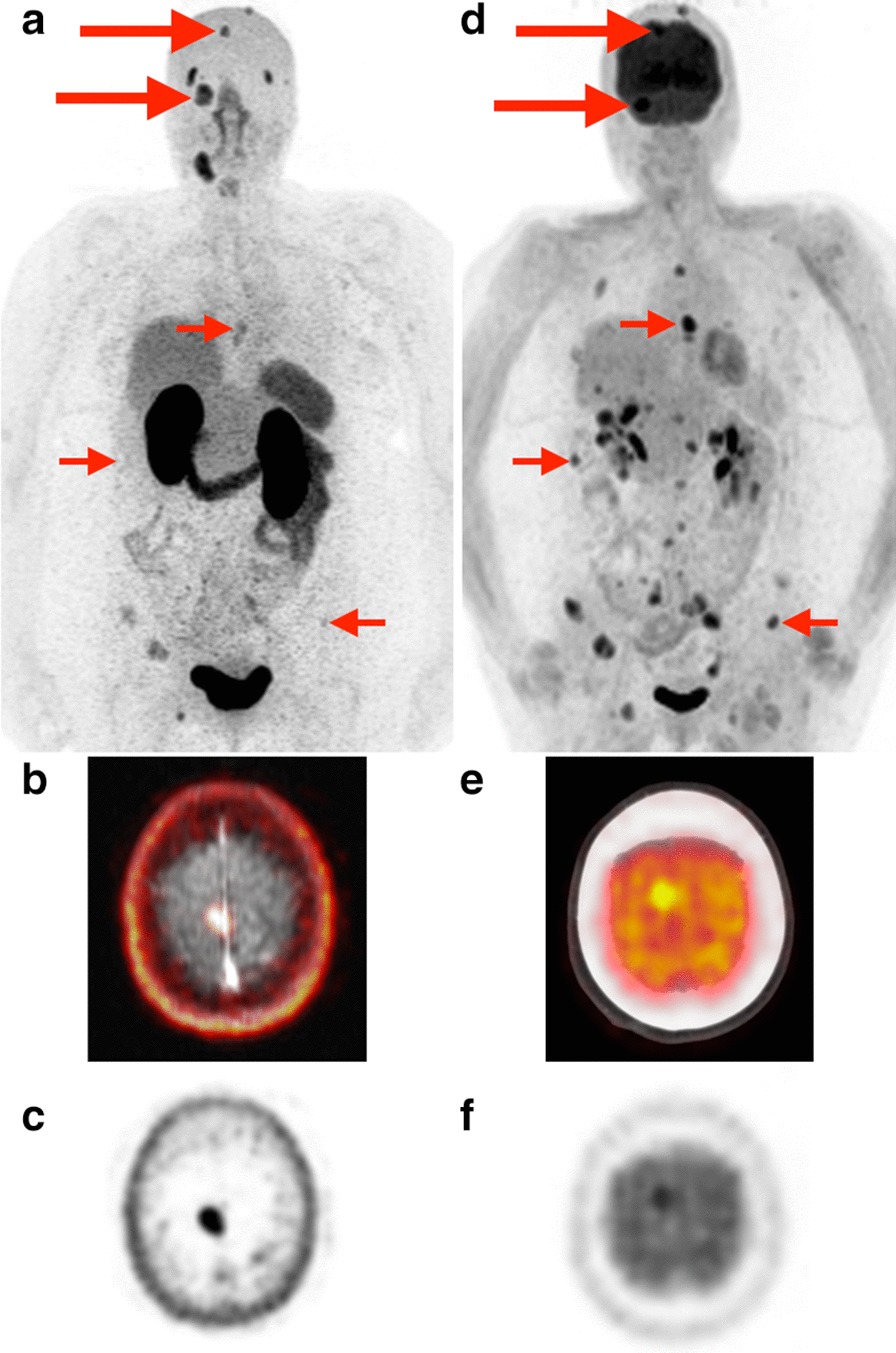
Dural and skull base metastases.[^68^Ga]Ga-PSMA-11 PET MIP (**a**) and 2-[^18^F]FDG PET MIP (**d**) in a patient (Patient 2) with Hurthle cell carcinoma. Dural and skull base metastases (large arrows) are better delineated on PSMA PET due to lack of background brain uptake (example: right falcine dural metastasis better seen on fused axial PSMA PET/MRI (**b**) and axial PSMA PET (**c**) than on fused axial FDG PET/CT (**e**) and axial FDG PET (**f**). Extensive metastatic disease involving the left adrenal gland, ribs, thoracolumbar spine, and pelvis is better seen on FDG PET in this patient

PSMA PET offered an advantage in visualization of intracranial metastases due to the lack of background brain uptake compared to FDG (Fig. 5). However, multiple extracranial metastatic lesions in the same patient were better seen on FDG. In addition, evidence of molecular heterogeneity was noted in some individual tumors. For example, Patient 10 with FTC had an expansile left iliac bone metastasis with a large soft tissue component (Additional file 1: Figure S2, C–J). While the soft tissue component was highly avid on both PSMA PET and FDG PET, the osseous component was much less avid. In contrast, the [^123^I]Iodide SPECT/CT of the same lesion demonstrated the reverse pattern, with no uptake in the soft tissue component and avid uptake in the osseous component.

Neck MRI findings included post-treatment changes (thyroidectomy, scarring, and/or lymph node dissection) in all patients, osseous metastases in 2/11 (PET avid), suspicious thyroid bed soft tissue (PET avid) in 1/11, “treated” thyroid bed soft tissue (PET avid, raising suspicion for residual disease) in 1/11, and cervical nodal metastases (only FDG avid, not PSMA) in 1/11.

No adverse events were reported.

## Discussion

In this study, PSMA PET was able to detect metastatic thyroid cancer, but at a lower rate than FDG PET. Furthermore, the degree of radiotracer avidity was heterogeneous between patients and, in some cases, within a given patient. In some cases for which FDG PET is not useful due to very low uptake, PSMA PET could serve as a more sensitive diagnostic PET agent (as seen in Patient 5 with FTC and Patient 6 with PDPTC), though our results suggest that PSMA avidity is not entirely predictable by cancer subtype or by FDG avidity.

The exploration of PSMA PET as a possible theranostic agent in thyroid cancer is in its infancy. The use of PSMA radioligand therapy (RLT) in DTC is beginning to be explored [15], but there are currently no accepted guidelines to determine eligibility for PSMA RLT in thyroid cancer. Even in prostate cancer, PSMA RLT eligibility criteria are heterogenous. Applying the same criteria to thyroid cancer patients may not be valid due to differences in PSMA uptake in prostate cancer (internalization into tumor cells) compared to thyroid cancer (cell surface binding in the tumor microvasculature); however, eligibility guidelines in the more well-studied prostate cancer population could serve as a useful starting point. Using one previously described set of criteria for PSMA RLT in prostate cancer [21] (lesion PSMA uptake greater than or equal to liver background, and no discordant PSMA negative/FDG positive lesions), 8/11 patients in our cohort could be eligible for PSMA RLT. Interestingly, this result was not predictable by differentiated versus dedifferentiated pathology of the primary; 5/7 DTC patients (71.4%) and 3/4 dedifferentiated subtype patients (75.0%) met criteria. However, using the eligibility criteria of a different PSMA RLT trial in prostate cancer (TheraP, [22]) (at least one lesion with SUVmax > 20, and no lesion > 10 mm with SUVmax < 10 on PSMA PET), no patients in our cohort would qualify for PSMA RLT. Given the lack of consensus on eligibility in prostate cancer, possible criteria in thyroid cancer are even less certain. de Vries et al. [15] administered PSMA RLT to two DTC patients deemed eligible based on most lesions being PSMA avid, with no strict SUV cutoffs. In order to develop appropriate eligibility criteria for PSMA RLT in the thyroid cancer population, there is a need for pharmacokinetic and dosimetric studies involving a large patient population. Since our results suggest that uptake is heterogeneous across thyroid cancer subtypes, future studies should involve both DTC and dedifferentiated subtypes.

Strengths of this study include its prospective design and comparative analysis to FDG PET, as well as the inclusion of multiple subtypes of thyroid cancer including both differentiated and dedifferentiated subtypes. Although RAI-refractory DTC has been imaged with PSMA PET [15, 23], anaplastic and poorly differentiated papillary thyroid cancer have not been as widely examined. Since dedifferentiated thyroid cancers are also RAI-refractory, this patient population merits evaluation of the utility of PSMA PET, if for no other reason than investigating the potential for PSMA RLT. Although our study was small, we found that the presence of dedifferentiated thyroid cancer did not necessarily preclude possible PSMA RLT eligibility. While these findings must be validated with a larger cohort, our results suggest that thyroid cancer subtype alone is not sufficient to predict suitability for PSMA PET imaging or possible PSMA therapy consideration.

The detection rate of PSMA and FDG PET in our cohort differed somewhat from some prior reports [23], but was similar with other previous findings [15, 24]. The largest prior study to date of ^68^Ga-PSMA PET in thyroid cancer involved 10 patients and excluded patients with dedifferentiated tumors [23]. That study found a DTC detection rate of 93.8% for PSMA PET (versus 45.9% in our study) and 81.8% for FDG PET (versus 94.6 in our study). In addition, reported lesion SUVmax values for DTC on PSMA PET (median SUVmax 31.4, range 4.9–101.8) differed markedly from those found in our study (median SUVmax 9.0, range 3.5–28.4). Although that study only included PTC and FTC (not HCC), the addition of HCC in our cohort is not sufficient to explain the observed difference in detection rate and median SUVmax. Our results were similar to a more recent study involving 5 DTC patients [15] which found a median lesion SUVmax of 3.6 (range 2.9–5.1) for cervical metastases and 4.1 (range 0.8–10.6) for distant metastases. Lütje et al. investigated PSMA PET in a cohort of six patients with iodine refractory, ^18^F-FDG positive patients, and found that 5 of 6 patients were positive on PSMA PET [24]. Similar to our own results, they found significant heterogeneity of PSMA uptake, with patients ranging from low to high degrees of uptake. Other cases of PSMA uptake in thyroid cancer have been reported, including differentiated thyroid cancer, follicular thyroid cancer [13], and anaplastic thyroid cancer [25, 26]. Our results complement the existing literature and demonstrate significant variability of PSMA uptake in thyroid cancer.

Uptake was heterogeneous within some patients, with some lesions only visible on PSMA and some only visible on FDG (though most lesions were visible to some degree on both). Intracranial lesions were easier to identify on PSMA PET due to lack of physiologic brain uptake compared to FDG. These findings suggest that PSMA PET may be a useful adjunct to FDG PET in the evaluation of metastatic thyroid cancer. PSMA avidity was not predicted by thyroid cancer subtype in our cohort, suggesting that cancer subtype should not dictate which metastatic thyroid cancer patients may benefit from PSMA PET.

### Limitations

Our study had multiple limitations. The first is the small size of our cohort. The desire to represent many different thyroid cancer subtypes resulted in a small number of patients within each subtype (the largest being PTC with *n* = 3). Although this broad representation may improve the generalizability of the findings overall, it significantly limits the conclusions that can be drawn about PSMA PET characteristics of individual subtypes. It also may explain why our findings in DTC were so different from at least one prior study as mentioned previously. Secondly, histopathologic confirmation was not available for most metastases; the presence and subtyping of disease was based on findings at thyroidectomy, and if applicable at cervical lymph node dissection. This may have introduced significant bias in detection rate calculation for both FDG PET and PSMA PET. In addition, thyroid cancer subtype determination was based on histopathology at the time of diagnosis, not at the time of scanning which in a few cases was years later. Because many thyroid cancers dedifferentiate over time (as evidenced by loss of radioiodine avidity), the originally determined subtype may not reflect the subtype at the time of scanning.

### Future directions

In order to more definitively characterize PSMA uptake in thyroid cancer, a larger study is needed with more cases of each thyroid cancer subtype. In particular, the inclusion of dedifferentiated thyroid cancer subtypes (PDPTC, ATC) should be considered. A potential application for which more data are needed is the administration of ^177^Lu-PSMA therapy for thyroid cancer patients who qualify based on imaging criteria. While ^68^Ga-PSMA uptake in the tumor may be considered a requirement for successful therapy, other factors such as volume of the target and retention rate are also important, particularly when using a probe which is not internalized to cancer cells. Building on an early pilot study of PSMA RLT involving 2 DTC patients [15], more thyroid cancer patients need to be treated in order to determine both safety and efficacy in this patient population.

## Conclusion

[^68^Ga]Ga-PSMA-11 PET may be complementary to 2-[^18^F]FDG PET for lesion visualization and may be useful for determining patient eligibility for PSMA radioligand therapy. Thyroid cancer subtype alone is not sufficient to predict PSMA uptake or PSMA radioligand therapy eligibility, as radiotracer uptake varies between patients and even within patients. Future studies should include a larger number of patients, including dedifferentiated thyroid cancer subtypes, and should involve administration of PSMA radioligand therapy.

## Supplementary information

**Additional file 1: Figure S1.** All differentiated PTC patients. A–B: Patient 1. C–D: Patient 7. E–F: Patient 9. Yellow circles indicate radiotracer-positive metastases. **Figure S2.** All FTC patients. A–B: Patient 5. C–D: Patient 10. Yellow circles indicate radiotracer-positive metastases. A left iliac metastasis with a large soft tissue component (E–J) demonstrated an inverse pattern of radioiodine uptake (K–M) compared to PSMA (E–G) and FDG PET (H–J). E–G: fused axial PSMA PET/MRI (E), axial PSMA PET (F), and axial T2-weighted MRI (G); H–J: fused axial FDG PET/CT (H), axial FDG PET (I), and axial CT (J); K–M: fused axial I-123 SPECT/CT (K), axial I-123 SPECT (L), axial CT (M). **Figure S3. **All HCC patients. A–B: Patient 2. C–D: Patient 11. Yellow circles indicate radiotracer-positive metastases. **Figure S4**. All PDPTC patients. A–B: Patient 3. C–D: Patient 6. Yellow circles indicate radiotracer-positive metastases. **Figure S5**. All ATC patients. A–B: Patient 4. C–D: Patient 8. Yellow circles indicate radiotracer-positive metastases.

## Data Availability

All included patients’ PSMA and FDG MIPs have been included under additional file figures.

## Abbreviations

ATC: Anaplastic thyroid cancer
DTC: Differentiated thyroid cancer
FDG: Fluorodeoxyglucose
FTC: Follicular thyroid cancer
HCC: Hurthle cell carcinoma
MRI: Magnetic resonance imaging
PDPTC: Poorly differentiated papillary thyroid cancer
PET: Positron emission tomography
PSMA: Prostate-specific membrane antigen
PTC: Papillary thyroid cancer
RLT: Radioligand therapy
