# Supplementary material for: Gallium-68 prostate-specific membrane antigen ([68Ga]Ga-PSMA-11) PET for imaging of thyroid cancer: a feasibility study
Source: EJNMMI Res. 2020 Oct 22;10:128. doi: 10.1186/s13550-020-00720-3 (PMC7581659; doi:10.1186/s13550-020-00720-3)
Supplement: Supplementary file 1 — Additional file 1: Figure S1. All differentiated PTC patients. A–B: Patient 1. C–D: Patient 7. E–F: Patient 9. Yellow circles indicate radiotracer-positive metastases. Figure S2. All FTC patients. A–B: Patient 5. C–D: Patient 10. Yellow circles indicate radiotracer-positive metastases. A left iliac metastasis with a large soft tissue component (E–J) demonstrated an inverse pattern of radioiodine uptake (K–M) compared to PSMA (E–G) and FDG PET (H–J). E–G: fused axial PSMA PET/MRI (E), axial PSMA PET (F), and axial T2-weighted MRI (G); H–J: fused axial FDG PET/CT (H), axial FDG PET (I), and axial CT (J); K–M: fused axial I-123 SPECT/CT (K), axial I-123 SPECT (L), axial CT (M). Figure S3. All HCC patients. A–B: Patient 2. C–D: Patient 11. Yellow circles indicate radiotracer-positive metastases. Figure S4. All PDPTC patients. A–B: Patient 3. C–D: Patient 6. Yellow circles indicate radiotracer-positive metastases. Figure S5. All ATC patients. A–B: Patient 4. C–D: Patient 8. Yellow circles indicate radiotracer-positive metastases. [file 13550_2020_720_MOESM1_ESM.docx]

Additional file 1: Figures


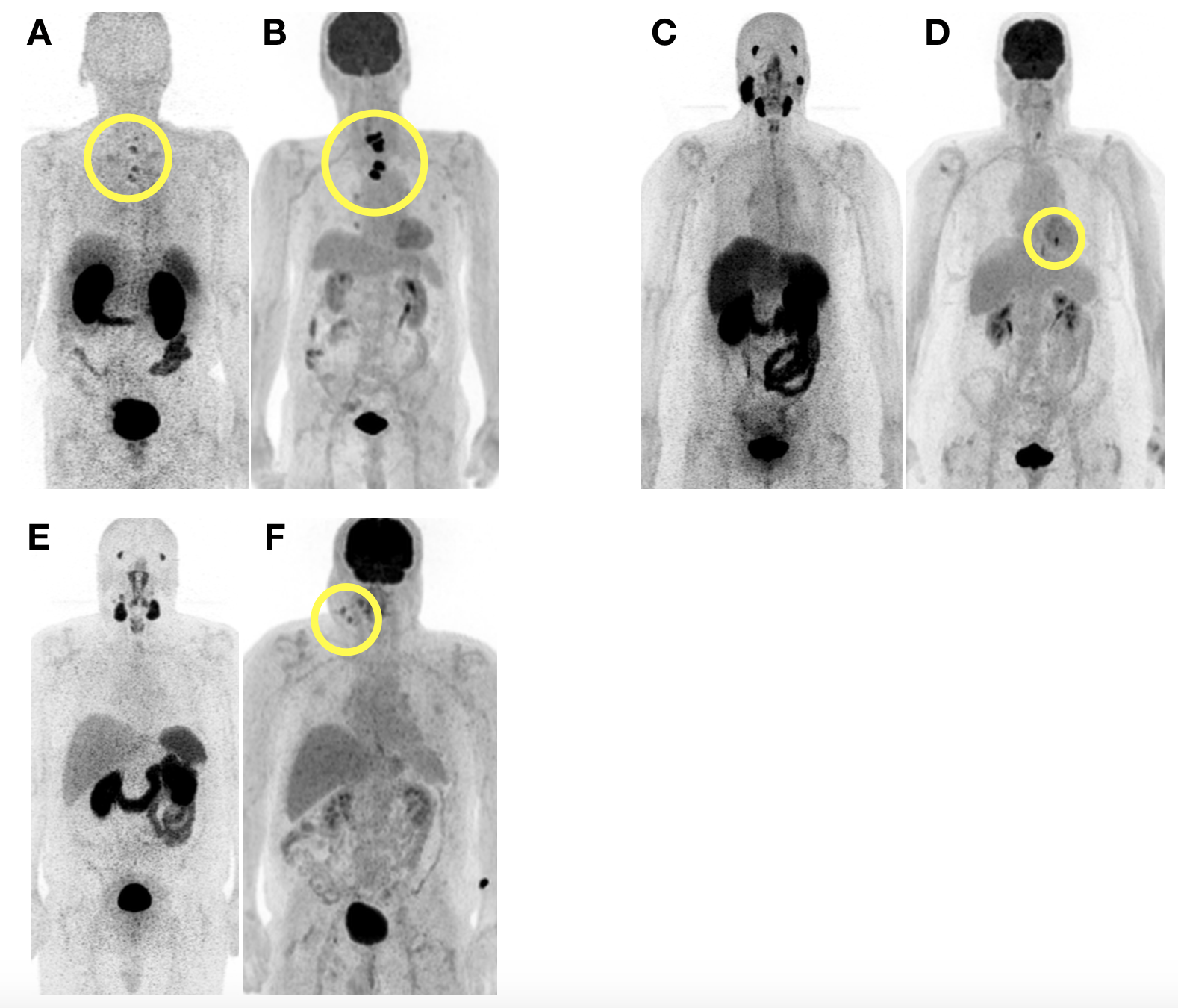
 **Figure S1**: All differentiated PTC patients. A-B: Patient 1. C-D: Patient 7. E-F: Patient 9. Yellow circles indicate radiotracer-positive metastases.


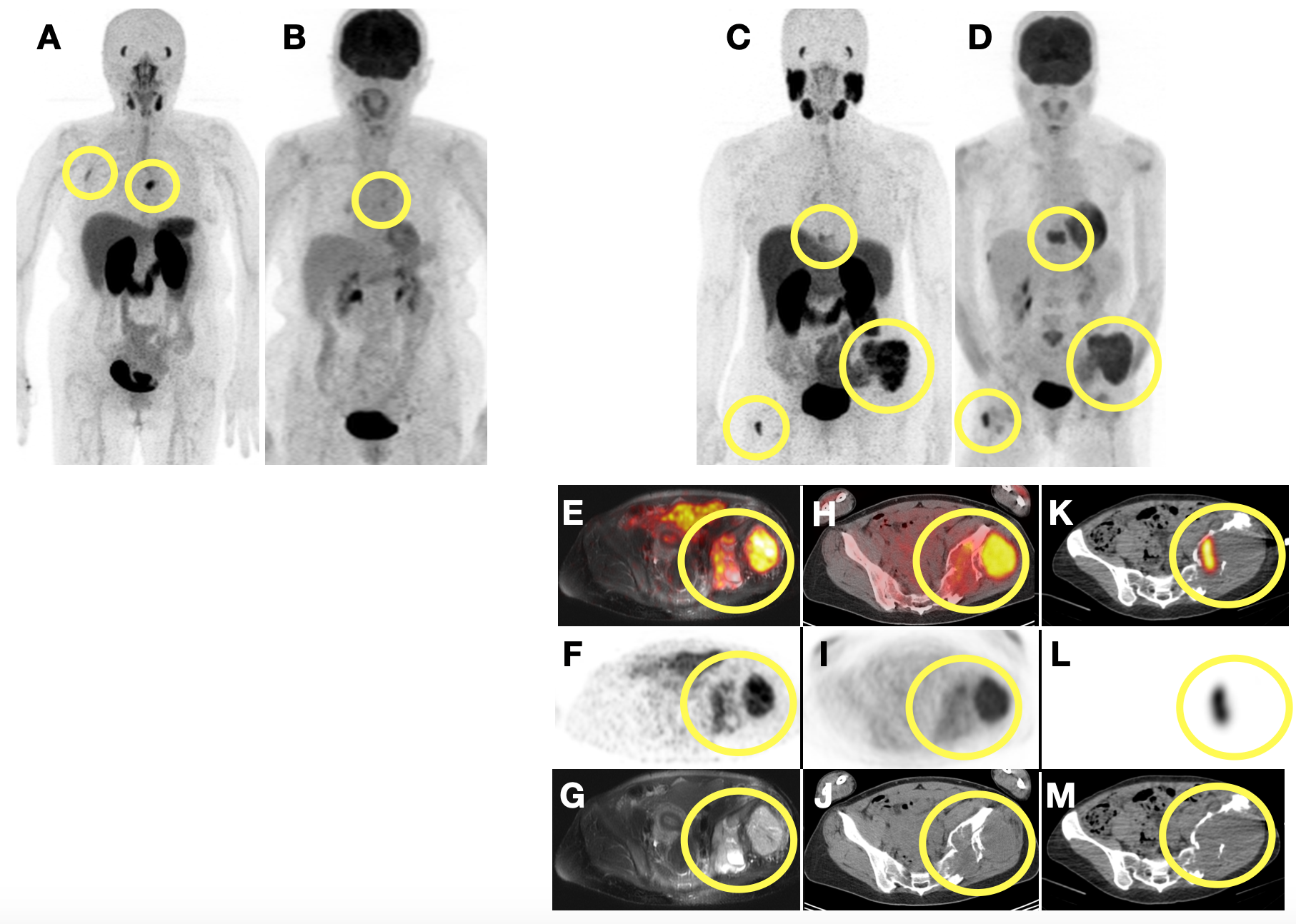


**Figure S2:** All FTC patients. A-B: Patient 5. C-D: Patient 10. Yellow circles indicate radiotracer-positive metastases. A left iliac metastasis with a large soft tissue component (E-J) demonstrated an inverse pattern of radioiodine uptake (K-M) compared to PSMA (E-G) and FDG PET (H-J). E-G: fused axial PSMA PET/MRI (E), axial PSMA PET (F), and axial T2-weighted MRI (G); H-J: fused axial FDG PET/CT (H), axial FDG PET (I), and axial CT (J); K-M: fused axial I-123 SPECT/CT (K), axial I-123 SPECT (L), axial CT (M).


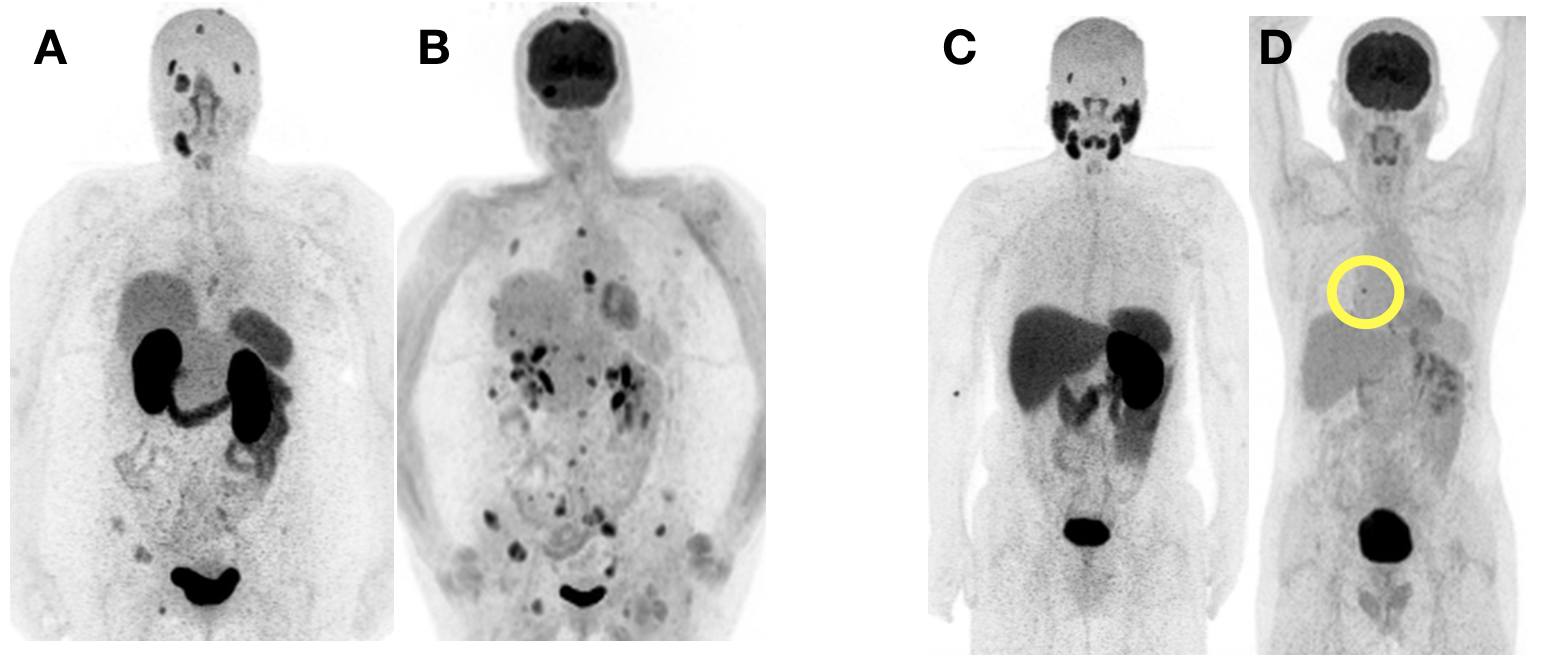


**Figure S3:** All HCC patients. A-B: Patient 2. C-D: Patient 11. Yellow circles indicate radiotracer-positive metastases.


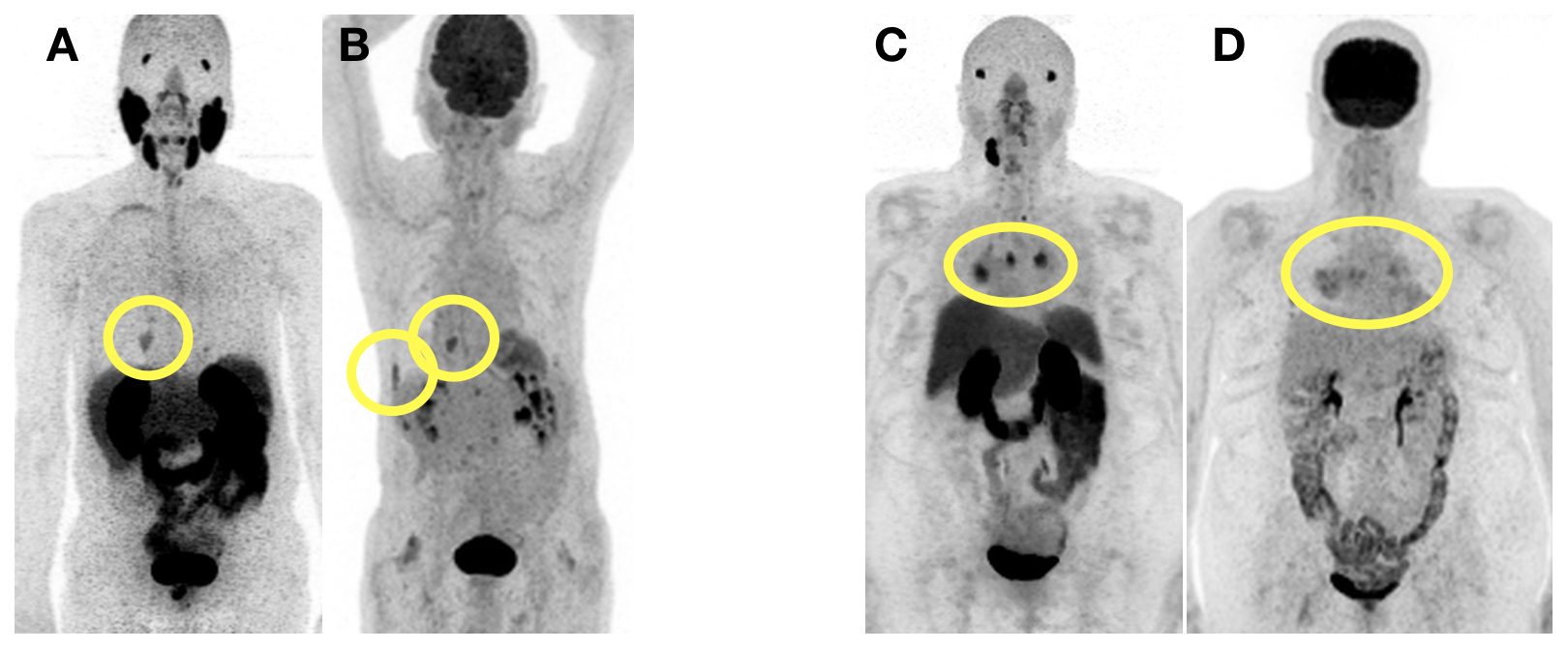


**Figure S4**: All PDPTC patients. A-B: Patient 3. C-D: Patient 6. Yellow circles indicate radiotracer-positive metastases.


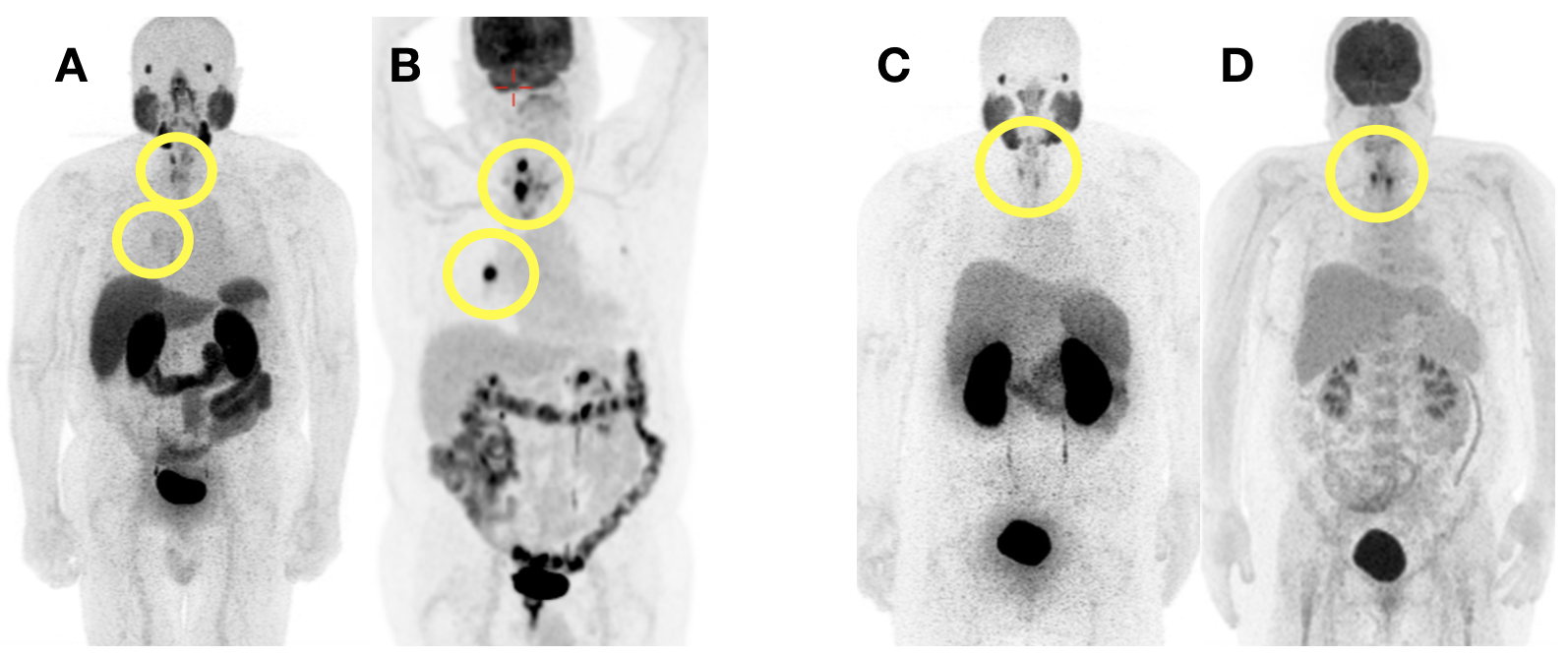


**Figure S5**: All ATC patients. A-B: Patient 4. C-D: Patient 8. Yellow circles indicate radiotracer-positive metastases.
